# Inter-Kingdom Signaling of Stress Hormones: Sensing, Transport and Modulation of Bacterial Physiology

**DOI:** 10.3389/fmicb.2021.690942

**Published:** 2021-10-06

**Authors:** Amine Mohamed Boukerb, Melyssa Cambronel, Sophie Rodrigues, Ouiza Mesguida, Rikki Knowlton, Marc G. J. Feuilloley, Mohamed Zommiti, Nathalie Connil

**Affiliations:** ^1^Laboratoire de Microbiologie Signaux et Microenvironnement EA 4312, Université de Rouen, Normandie Université, Évreux, France; ^2^EA 3884, LBCM, IUEM, Université de Bretagne-Sud, Lorient, France; ^3^MRC Laboratory of Molecular Biology, Cambridge, United Kingdom

**Keywords:** stress hormones, catecholamines, sensing, transport, bacterial physiology

## Abstract

Prokaryotes and eukaryotes have coexisted for millions of years. The hormonal communication between microorganisms and their hosts, dubbed inter-kingdom signaling, is a recent field of research. Eukaryotic signals such as hormones, neurotransmitters or immune system molecules have been shown to modulate bacterial physiology. Among them, catecholamines hormones epinephrine/norepinephrine, released during stress and physical effort, or used therapeutically as inotropes have been described to affect bacterial behaviors (i.e., motility, biofilm formation, virulence) of various Gram-negative bacteria (e.g., *Escherichia coli, Salmonella enterica* serovar Typhimurium, *Pseudomonas aeruginosa, Vibrio* sp.). More recently, these molecules were also shown to influence the physiology of some Gram-positive bacteria like *Enterococcus faecalis.* In *E. coli* and *S. enterica*, the stress-associated mammalian hormones epinephrine and norepinephrine trigger a signaling cascade by interacting with the QseC histidine sensor kinase protein. No catecholamine sensors have been well described yet in other bacteria. This review aims to provide an up to date report on catecholamine sensors in eukaryotes and prokaryotes, their transport, and known effects on bacteria.

## Introduction

Stress is a complex event that impacts the homeostasis of the whole organism. In cases of stress, the concentration/release of stress hormones/catecholamines may be raised by 20–40 times the physiological values and can reach 0.17–0.54 μg per minute. In some organs, such as the spleen or the gut, they can lead to local concentrations of up to 0.1–1 mM (Felten and Olschowka, 1987; Bergquist et al., 1998). This is caused by discharge from synaptic vesicles at noradrenergic nerve endings. Catecholamines (dopamine, norepinephrine, and epinephrine) are found throughout the plant and animal kingdoms (Akula and Mukherjee, 2020). In mammals, they can stimulate lipolysis and glycogenolysis and mobilize energy more rapidly than cortisol, as the latter’s activity requires transcription of genes.

**FIGURE 1:**
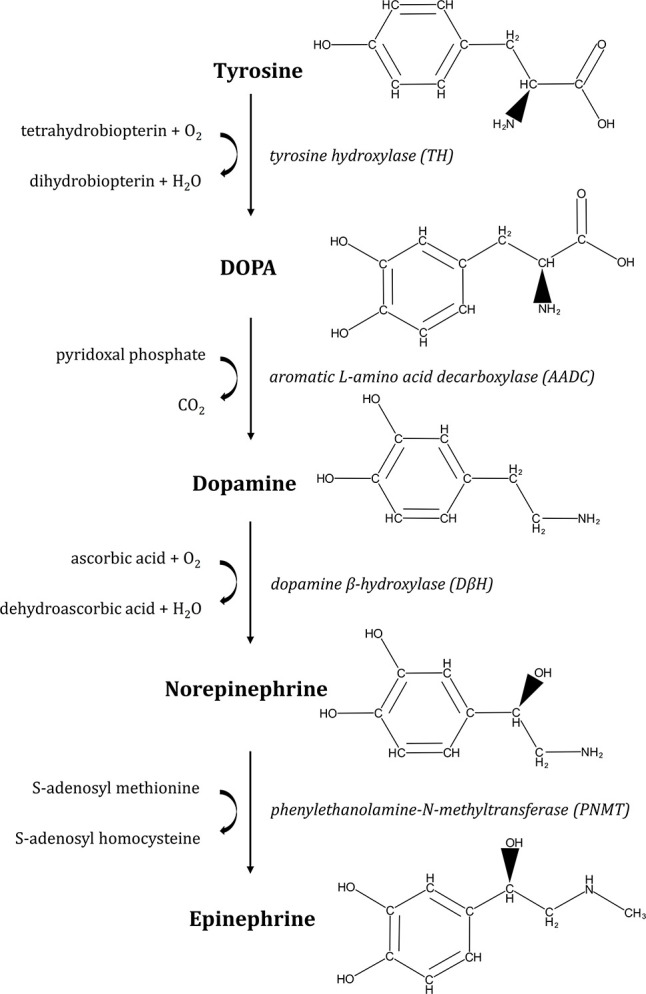
Pathway of epinephrine and norepinephrine biosynthesis. Synthesis of catecholamines starts with conversion of L-tyrosine to L-dopa by tyrosine hydroxylase (TH). Then, L-dopa is processed to dopamine by L-aromatic amino acid decarboxylase (AADC), from where norepinephrine is formed by dopamine-β-hydroxylase (DβH). Finally, epinephrine is synthesized by addition of a methyl group to norepinephrine by phenylethanolamine-*N*-methyltransferase (PNMT). In mammals, catecholamines are synthesized from L-Dopa, obtained from dietary sources (the amino acids tyrosine and phenylalanine).

Catecholamines are organic nitrogen compounds derived from the amino acid L-tyrosine (Figure 1). The first step in the biosynthesis of these molecules is the hydroxylation of L-tyrosine to L-dopa (L-dihydroxy-phenylalanine) by tyrosine hydroxylase. L-dopa is then decarboxylated to dopamine by Dopa-decarboxylase. This hormone is successively converted to norepinephrine (NE) and then epinephrine (Epi) by dopamine β-hydroxylase and phenylethanolamine-*N*-methyltransferase, respectively.

Intestinal expression of tyrosine hydroxylase was found upregulated in response to surgical perforation of the bowel and gut-derived sepsis in rats (Zhou et al., 2004). Subsequently, high levels of NE were detected in their fecal pellets. Catecholamines can act as hormones or neurotransmitters. They are synthesized by the cells of the adrenal medulla and by the postganglion neurons of the orthosympathetic nervous system. Epi acts as a neurotransmitter in the central nervous system and as a hormone in the bloodstream. NE is primarily a neurotransmitter in the peripheral sympathetic nervous system but is also found in the blood. Dopamine is an essential neurotransmitter in the motivation and reward system.

In the human body, catecholamines act on almost all tissues and exert numerous activities at the cardiovascular, metabolic, endocrine, and neuronal levels. They also affect the intestinal barrier and immunity (Lymperopoulos et al., 2008). The action of catecholamines takes place after binding to specific receptors located on the cell membranes of target tissues: the α- and β-adrenergic receptors. The effects of catecholamines have long been studied only in humans without considering their possible impact on the microbiota. However, prokaryotes and eukaryotes have been intimately cohabiting for a very long time. In the gut and other tissues with contact to the external world via epithelial surfaces, catecholamines can cross the epithelial border and interact with microorganisms living in those ecological niches. In the colon, NE can reach a concentration of about 50 ng/g luminal content. Thus, bacteria have been in contact with the host hormones and have been able to develop complex interactions. In 1992, Lyte and Ernst were the pioneers who evaluated the effects of stress hormones on bacterial growth and introduced for the first time the concept of microbial endocrinology, a bi-directional interaction between human neuroendocrine factors and microorganisms (Lyte and Ernst, 1992; Sharaff and Freestone, 2011). It was subsequently shown that Epi/NE stimulates the growth of Gram-negative enteric pathogens of various genera (e.g., *Salmonella*, *Shigella*, *Yersinia*, *Vibrio*, and *Campylobacter*) as well as pathotypes of *E. coli* such as EHEC (Enterohemorrhagic *E. coli*) and ETEC (Enterotoxigenic *E. coli*). This new concept may help to understand how stress influences susceptibility to infection (Freestone et al., 2008). Since then, many studies have shown that catecholamines can have various stimulating effects on numerous Gram-negative and Gram-positive bacterial pathogens (i.e., growth, motility, biofilm formation, adhesion, cytotoxicity/virulence), and sensor systems allowing to perceive these molecules have been discovered for some bacterial species.

## Sensing of Catecholamines

### Eukaryotic (Human) Sensors of Catecholamines: Adrenergic Receptors

**FIGURE 2:**
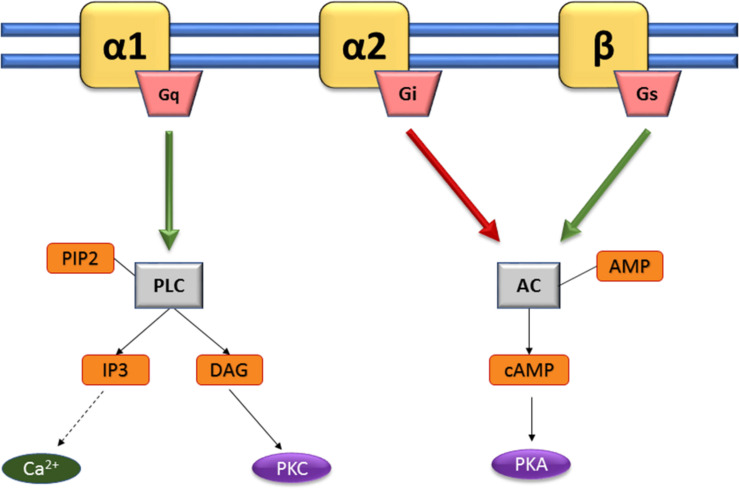
Activating pathways of adrenergic sensors. Catecholamines activate various cellular signal transduction by binding to α 1-, α 2-, and β-adrenoreceptors (yellow). The α1-receptor is coupled with Gq protein, allowing activation of kinase protein C (PKC) and increase of intracellular concentration of Ca2+, through the triphosphate inositol (IP3)/diacylglycerol (DAG) pathway. Activation of this pathway results from the cleavage of phosphatidylinositol-4,5-bisphosphate (PIP2) in IP3 and DAG thanks to phospholipase C (PLC). α-2 and β-receptors are coupled to Gi and Gs, respectively. In both cases, cAMP (cyclic adenosine monophosphate) is increased or decreased depending on the stimulation (Gs) or inhibition (Gi) of adenylate cyclase (AC), leading to the activation of kinase protein A (PKA). Adapted from Andreis and Singer (2016).

Pharmacological classification of adrenergic receptors (AR) as α- and β-adrenergic receptors (Table 1) was first described in 1948 (Ahlquist, 1948). This classification was established according to their pharmacological properties and physiological effects (Perez, 2006). The α-adrenergic receptors are mainly involved in excitatory functions (vasoconstriction, uterine contraction, contraction of the nictitating membrane, pupillary dilation) whereas β-receptors are more commonly related to inhibitory responses (vasodilatation, inhibition of uterine contraction, myocardial stimulation). The α-receptor group is subdivided in two sub-groups: α-1 and α-2 (Berthelsen and Pettinger, 1977). Each of them is composed of three subtypes, α-1A, α-1B, and α-1D (Ford et al., 1994), or α-2A/D, α-2B, and α-2C (Bylund, 1988; Hieble et al., 1995). β-receptors are also subdivided in three subtypes: β-1, β-2, and β-3 (Lands et al., 1967a,b; Emorine et al., 1989). The first cloned and characterized drug receptor was the β-2 AR (ADRB2) that binds Epi (Dixon et al., 1986). NE has relatively higher binding affinity for α- ARs and β-1/3 ARs, but a lower affinity for β-2 AR. All these receptors belong to the group of G Protein-Coupled Receptors (GPCRs), the largest family of cell-surface proteins involved in signal transduction (Rosenbaum et al., 2009). They contain seven transmembrane domains (Strosberg, 1993), and are linked to a GTP-binding regulatory G protein. The G protein is composed of three subunits: α, β, and γ. The α subunit determines the signal transduction pathway which will modulate the activity of second messengers (Marinissen and Gutkind, 2001). Generally, G proteins can be divided into Gi, Gs, Gq/11, and G12/13 subfamilies according to their α subunits. Catecholamine receptors have been defined using highly selective receptor agonists and antagonists in functional pharmacological investigations, delivering information on their affinities (Kd, dissociation constant). However, such studies do not reflect the biological activity of the ligand-receptor interaction, which is an entity functionally coupled to intracellular signal transduction pathways. Thus, new strategies are now used on isolated cells and transgenic animals based on molecular cloning and structure-function analysis to encounter such limitations, providing precious data on selective antagonism, rank order of ligand potency and stereospecificity. Catecholamines activate various cellular signal transduction by binding to these receptors, activating the G proteins which can then modulate effectors such as adenylate cyclase or phospholipase C (PLC) (Figure 2). The α1-receptor is coupled with G_*q*_ protein, allowing activation of kinase protein C (PKC) and increase of intracellular concentration of Ca^2+^, through the triphosphate inositol (IP3)/diacylglycerol (DAG) pathway. Activation of this pathway results from the cleavage of phosphatidylinositol-4,5-bisphosphate (PIP2) in IP3 and DAG thanks to phospholipase C (PLC). α-2 and β-receptors are coupled to G_*i*_ and G_*s*_, respectively. In both cases, cAMP (cyclic adenosine monophosphate) is increased or decreased depending on the stimulation (G_*s*_) or inhibition (G_*i*_) of adenylate cyclase (AC), leading to the activation of kinase protein A (PKA). Variations in the concentrations of the intracellular secondary messengers lead to physiological modifications (e.g., vasoconstriction, uterine contraction or pupillary dilatation) (Table 1).

**TABLE 1:** Adrenergic receptors and physiological effects.

Subtypes	G proteins	Intracellular messengers	Target organs	Physiological effects
α1	Gq	Increase in PLC and IP3	Uterine	Contraction
			Vascular smooth muscles	Contraction
			Blood vessels	Constriction
			GI sphincter	Increase in tone
			Urinary sphincter	Increase in tone
			Pupillary radial muscle	Contraction (mydriasis)
			Pilomotor smooth muscle	Contracts (erects hair)

α2	Gi	Decrease in cAMP	Presynaptic nerves	
			Adrenergic and cholinergic nerves terminals	Inhibit transmitter release
			Platelets	Stimulate aggregation
			Some vascular smooth muscle	Contraction
			Fat cells	Inhibit lipolysis
			Pancreatic B cells	Inhibit insuline release
			Ciliary epithelium	Reduction of humor secretion

β1	Gs	Increase in cAMP	Heart	Stimulates rate and force
			Kidney	Stimulates renin release

β2	Gs	Increase in cAMP	Liver	Stimulates glycogenolysis
			Pancreatic B cells	Stimulates insulin release
			Skeletal muscle	Contraction
			Heart	Stimulates rate and force
			Ciliary epithelium	Increases of humor secretion
			Airways, uterine and vascular smooth muscle	Relaxes
			Uterine	Inhibit contraction

β3	Gs	Increase in cAMP	Adipose tissues (Fat cells)	Stimulates lipolysis

Several antagonists can bind to the adrenergic receptors with a high affinity, and competitively block the effects of ligands. For example, α-1 and β receptors can be respectively inhibited by phentolamine and propranolol. The latter is used in medical settings for hypertension as a β-blocker with an antihypertensive role (Lewis, 1976).

### Prokaryotic Sensors of Catecholamines

As aforementioned, the study of microbiota response to eukaryotic signaling molecules and resulting behavior of the host is known as microbial endocrinology, highlighted by Lyte and Ernst (1992), but first evidence of interaction between bacterial pathogens and the host were described as early as 1930 (Renaud and Miget, 1930) when the syringe used to inject Epi was not correctly sterilized, leading to gas gangrene. A similar case was also described by Cooper (1946). Following these discoveries, many studies were carried out to understand the effect of Epi on bacteria. Various physiological effects of catecholamines were observed (see below) and sometimes the presence of a bacterial sensor for these molecules has been proposed.

**FIGURE 3:**
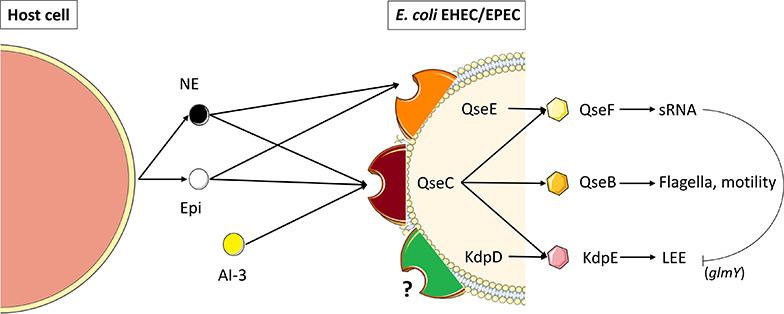
Catecholamine sensing and signal transduction in enterohemorrhagic and enteropathogenic *E. coli* (EHEC/EPEC). QseC is an adrenergic sensor kinase that autophosphorylates on detection of NE, Epi and AI-3 and transfers the phosphate moiety to its cognate response regulator QseB, thereby activating transcription of the flagellar regulon (Clarke et al., 2006). Transcription of genes encoding a second two-component system (QseEF) is sensitive to NE and Epi and is implicated in small RNAs expression (Reading et al., 2009). Kinase activity in QseC is promiscuous and can activate two additional non-cognate response regulators, KdpE and QseF. QseC also activates the locus of enterocyte effacement (LEE), through KdpE, which is inhibited through sRNAs (*glmY*) that are modulated by QseF. (Adapted from Ellermann and Sperandio, 2020). KdpD, a transmembrane protein showing partial homology with QseC may also act as a catecholamine receptor (Borrel et al., 2019).

The main signaling transduction systems in bacteria are the two-component systems. In these systems, the sensor for environmental cues is a histidine kinase, which upon autophosphorylation transfers its phosphate to an aspartate residue in the response regulator, which is usually a transcription factor that is activated by phosphorylation. Clarke et al. (2006) showed that the QseC sensor kinase is a bacterial receptor for the host Epi/NE (Figure 3). QseC is part of the QseBC system, initially described as a two-component system regulated by quorum sensing and involved in regulation of flagella and motility in EHEC. Sperandio et al. (2003) showed that Epi was sensed by EHEC serotype O157:H7 via QseC, and activated EHEC virulence regulators like *ler*, the regulator of the Locus of Enterocyte Effacement (LEE) which is a pathogenicity island that contains 41 genes. Most of them are organized in five major operons, coding for a type III secretion system (responsible for attaching and effacement lesion in the large intestine cells), in addition to an adhesin (intimin) and its receptor. QseC was found to activate the LEE operons through KdpE and QseF-regulated sRNAs, and suggested to be the sensor for LuxS-dependent autoinducer-3 (AI-3) and Epi (Figure 3). Rasko et al. (2008) showed that exposure of EHEC strain 86-24 to 50 μM Epi increased the expression of the *ler* gene by 1.5-fold. Recently, the description of the complete structure of AI-3 uncovered the most active molecule within AI-3 family being a new pyrazinone-type of metabolite (Kim et al., 2020). QseC possesses a periplasmic domain with a sensor domain, two transmembrane domains and a kinase domain, while the response regulator QseB contains a receptor domain as well as a helix-turn-helix (HTH) domain. Activation of QseC leads to the transfer of phosphate to the response regulator QseB, which then regulates its own transcription, as well as the transcription of flagella and motility genes (Clarke and Sperandio, 2005a,b). In addition, an osmosensitive K^+^ channel histidine kinase, named KdpD, was found sharing partial homology with QseC, suggesting its possible role as a catecholamine receptor, which needs to be elucidated (Figure 3). Multiple studies using *E. coli* EHEC and UPEC (uropathogenic) isolates have demonstrated that *qseC* mutants have decreased motility (Sperandio et al., 2003; Hughes et al., 2009; Kostakioti et al., 2009; Hadjifrangiskou et al., 2011; Guckes et al., 2013). An *E. coli* O157:H7 *qseC* mutant presented pleiotropic phenotypes including virulence attenuation, metabolic dysregulation and decreased motility (Kostakioti et al., 2009; Hadjifrangiskou et al., 2011). Interestingly, an *E. coli* O157:H7 *qseBC* mutant has a similar motility phenotype compared to wild-type O157:H7 (Sharma and Casey, 2014a,b). However, in response to 50 μM NE, they observed a significant increase in the motility of *E. coli* O157:H7 *qseC* and *qseBC* mutants. These results suggest that in addition to QseC, other regulatory systems sense and respond to Epi and NE in *E. coli*. As such, QseC seems to be involved in an inter-kingdom communication by sensing both bacterial quorum sensing molecules (i.e., AI-3) and hormones secreted by the host (i.e., Epi and NE). Another two-component system found in *E. coli* EHEC, named QseEF (Figure 3) was shown to belong to the signaling cascade Epi/NE/AI-3 (Reading et al., 2007). The transcription of *qseE* can be activated by the sensor QseC, after recognition of Epi. QseE can also be activated by the presence of Epi (Figure 3) but not by AI-2 or AI-3 (Reading et al., 2009). An *in silico* screening of the sensing-domain of QseC revealed a high degree of conservation within several bacterial species (Clarke et al., 2006), encompassing *S. enterica* serovar Typhimurium in which it may participate in Epi/NE signaling. Furthermore, it has been shown that in absence of *qseC* in this bacterium, NE was still able to activate the transcription of some genes, meaning that QseC is not the only sensor of Epi/NE in *S. enterica* serovar Typhimurium (Moreira et al., 2010). During the same period, other alternative sensor systems for Epi/NE have been described in *Salmonella* such as BasRS and CpxAR (Karavolos et al., 2008, 2011). To our knowledge, no other sensors for catecholamines have been described lately in bacteria, except two recent works on *Cutibacterium acnes* (former *Propionibacterium acnes*), and *E. faecalis* suggesting the role of KdpD and VicK (WalK), respectively, as putative adrenergic receptors (Borrel et al., 2019; Cambronel et al., 2020). The latter authors identified VicK as the closest protein to QseC with 29% identity and 46% similarity values. Structure modeling and molecular docking of VicK corroborated its possible interactions with Epi and NE, with binding energies of –4.08 and –4.49 kcal/mol, respectively.

Eukaryotic adrenergic antagonists were used in some studies and found to be able to block the physiological effects of catecholamines in bacteria (see below). LED209 [*N*-phenyl-4-(3-phenylthioureido) benzenesulfonamide], a bacterial inhibitor of QseC has also been identified through a high-throughput screening of a library of 150,000 small organic compounds and subsequent lead optimization (Rasko et al., 2008). This inhibitor has a unique mode of action by acting as a prodrug scaffold to deliver a warhead that allosterically modifies QseC, impeding virulence in several Gram-negative pathogens (Curtis et al., 2014).

## Transport of Catecholamines

### Transport in Eukaryotes

The monoamines, including the catecholamines (Epi, NE, dopamine), are a group of important neurotransmitters and neurohormones (Duan and Wang, 2010) that regulates a wide array of physiological, behavioral, cognitive, and endocrine functions in central and peripheral nervous systems (Carlsson, 1987; Greengard, 2001). The actions of released monoamine neurotransmitters are terminated by plasma membrane transporters that actively remove them from the extracellular space (Duan and Wang, 2010). Two distinct transport systems, named uptake1 and uptake2, are responsible for the clearance of monoamines in eukaryotic cells (Gründemann et al., 1998; Eisenhofer, 2001). Uptake1 consists of Na^+^ and Cl^–^ dependent, high-affinity transporters while the uptake2 was originally characterized as a Na^+^ and Cl^–^ independent, low-affinity, high-capacity transport system in peripheral tissues such as heart and smooth muscle cell (Iversen, 1971; Bönisch et al., 1985; Eisenhofer, 2001). The uptake2 system has also been found in various brain areas like cortex and striatum (Hendley et al., 1970; Wilson et al., 1988). In addition, studies on transport of monoamines in the brains, of serotonin (5-HT = 5-hydroxytryptamine), dopamine, and NE transporters knockout mice, demonstrated the existence of heterologous uptake of monoamines that cannot be exhaustively explained by the promiscuity of the abovementioned neuronal high-affinity monoamine transporters (Sora et al., 2001; Wayment et al., 2001; Moron et al., 2002). The plasma membrane monoamine transporter (PMAT) and organic cation transporter 3 (OCT3) are the two most prominent low-affinity, high-capacity (uptake2) transporters for catecholamines (Duan and Wang, 2010). These authors demonstrated that hPMAT (human plasma membrane monoamine transporter) is the major uptake2 transporter for serotonin and dopamine in the central nervous system, whereas hOCT3 (human organic cation transporter 3) represents the major uptake2 transporter for histamine, NE, and Epi in peripheral organs. The PMAT transports dopamine and serotonin with Km values in the high micromolar range while histamine, Epi, and NE are transported with Km values in the low millimolar range (Engel et al., 2004; Engel and Wang, 2005). The PMAT is widely distributed throughout the brain with the highest expression in the forebrain cortex, olfactory tubercle, hippocampus, and cerebellum (Dahlin et al., 2007; Vialou et al., 2007). PMAT expression has been detected in diverse populations of neurons including pyramidal neurons, interneurons, granular neurons, and Purkinje cells while no significant expression was measured in astroglial cells (Dahlin et al., 2007).

### Transport in Prokaryotes

Information about putative entrance/transport of catecholamines in bacteria is scarce and not well understood. Lyte and Brown (2018) hypothesized that probiotics belonging to the genus *Lactobacillus* express uptake systems of biogenic amines, based on the observations of Yernool et al. (2004) and Yamashita et al. (2005), who demonstrated that bacterial transporters analogous to glutamate and leucine were expressed in bacteria. To test their hypothesis, Lyte and Brown have examined monoamine uptake in *Lactobacillus* biofilms using fluorescent probes for membrane amine transport (Lyte and Brown, 2018). The results of this study suggested that some lactobacilli biofilms express functional homologs of PMAT and OCT, which could take up and potentially deliver bioactive amines to nearby microbes or host cells in the intestinal tract. Bacterial analogs of biogenic amine transporters may contribute to the ability of the microbiota-gut-brain axis to influence brain function and ultimately behavior (Lyte, 2014; Sharon et al., 2016). It remains to be determined whether other bacterial species can express these biogenic amine transporters.

The putative conversion of catecholamines by bacteria has also been suggested (Toulouse et al., 2019; Reiske et al., 2020). Epi is converted by the pathogen *V. cholerae* to adrenochrome in the course of respiration. Superoxide produced by the respiratory Na^+^ translocating NADH: quinone oxidoreductase (NQR) act as an electron acceptor in the oxidative conversion of Epi to adrenochrome.

## Modulation of Bacterial Physiology

### Effect on Growth and Capture of Iron

*In vitro* antimicrobial activity of catecholamines have been observed by Cuvas Apan et al. (2016) and Kesici et al. (2019). In fact, Cuvas Apan et al. (2016) found that saline dilutions of Epi, NE, and dopamine at clinically used concentrations decreased microbial growth of *Staphylococcus aureus, Staphylococcus epidermidis*, and *Candida albicans*, whereas *E. coli* and *P. aeruginosa* were more resistant. Despite this, most of the studies that were carried out have shown that catecholamines generally had no impact on bacterial growth when the bacteria were cultivated in rich media. On the contrary, experiments performed in minimal media or in low-iron media like serum-SAPI showed an increase of bacterial growth in presence of catecholamines for several bacteria (Table 2). For example, NE can enhance the growth of *S. enterica* serovar Typhimurium in serum-SAPI minimal medium (Freestone et al., 1999; Williams et al., 2006), suggesting that additional biosynthetic pathways would be modulated besides the iron utilization and transport genes. Using microarray-based transcriptional analysis on *S. enterica* serovar Typhimurium grown in serum-SAPI medium with or without 2 mM NE, Bearson et al. (2008) found an increase in transcription of genes involved in amino acid biosynthesis, cofactor biosynthesis, energy metabolism, central intermediary metabolism and synthesis of transport and binding proteins. Thus, to take advantage of the increased availability of iron provided by NE in serum-SAPI minimal medium, *S. enterica* serovar Typhimurium modulates the biosynthesis of multiple cellular pathways to increase its growth rate. In fact, iron is an essential element for almost all organisms, as an indispensable enzymatic co-factor in many cellular processes; and most bacteria require micromolar levels of bioavailable iron for optimal growth (Ratledge and Dover, 2000). During infection, a coordinated host response limits the availability of iron to microbes and restricts the replication of invading pathogens (Ganz and Nemeth, 2015). Iron sequestration is mainly due to the mammalian ferric-iron-binding proteins transferrin in serum and lactoferrin in mucosal secretions (Mietzner and Morse, 1994). A strategy that bacteria often employ to collect essential iron is the production and utilization of siderophores, that possess high affinity for ferric iron (Ratledge and Dover, 2000). The catechol core found in many siderophores is also present in the stress-related hormones of the catecholamine family. Thus, many studies which show the effect of catecholamines on bacterial growth, were carried out using a serum-like medium containing transferrin (Tf) and lactoferrin (Lf). This medium more appropriately mimics a stressful and bacteriostatic environment for the bacteria and would therefore more closely resemble the conditions that the bacteria might encounter within the host (Burton et al., 2002; Freestone et al., 2003; Lyte, 2004). Sandrini et al. (2010) showed that Epi, NE, and dopamine formed a complex with the ferric iron Fe (III) present in Tf and Lf. The catechol nucleus of catecholamines will then bind the iron sequestered by Tf and Lf, reducing their affinity with them. Thus, the presence of catecholamines would stimulate growth of bacteria by making the iron contained in Tf and Lf available for them. However, the exact mechanism by which iron is incorporated into the bacteria in the presence of catecholamines remains unclear. One study has implicated the porin proteins OmpA and OmpC from several enteropathogens in transferrin binding and transferrin dependent iron uptake (Sandrini et al., 2013). The authors showed that the presence of OmpA may increase growth of bacteria in the presence of some catecholamines. The assimilation of the siderophore/iron complex through specific Ton-B dependent receptors has also been suggested (Ratledge and Dover, 2000). For example, in *P. aeruginosa*, there are at least 34 Ton-B dependent or putative receptors, including those involved in the reuptake of pyoverdine and pyochelin, the two main siderophores (Cornelis and Bodilis, 2009), and it has been shown that in presence of NE, the expression of the genes coding for the synthesis of these siderophores are decreased (Li et al., 2009). Additionally, Perraud et al. (2020) demonstrated that catecholamine neurotransmitters (dopamine, L-DOPA, Epi, and NE) can act as siderophores, chelating iron and efficiently bringing it into *P*. *aeruginosa* cells via the PiuA and PirA TonB-dependent transporters (TBDTs). These authors found that PiuA exhibited more pronounced specificity for dopamine uptake than for NE, Epi, and L-DOPA, whereas PirA specificity appeared to be higher for L-DOPA and NE. Similar mechanisms were suggested in other Gram-negative bacteria. Indeed, in *E. coli* (Burton et al., 2002; Freestone et al., 2003) and *Salmonella* (Williams et al., 2006; Dichtl et al., 2019) siderophore synthesis and uptake systems appeared also to be integral elements in the mechanism by which stress-related neuroendocrine hormone induce growth. *Salmonella enterica* produces enterochelin which bioavailability for bacterial iron acquisition is reduced by the mammalian siderocalin in addition to the affinity of enterochelin for lipid membranes. *Salmonella* glucosylation of enterochelin to salmochelin by IroB reduces both membrane affinity and siderocalin binding properties (Hantke et al., 2003). The increased bioavailability of salmochelin compared to enterochelin facilitates iron acquisition from transferrin to support pathogen growth. Due to their siderophore-like properties, Epi/NE accelerate bacterial iron acquisition and therefore can enhance pathogen growth in iron-limited environments. In *Bordetella bronchiseptica*, NE has been described to mediate acquisition of transferrin iron (Anderson and Armstrong, 2008; Armstrong et al., 2012). In *Actinobacillus pleuropneumoniae*, mutation of the gene encoding TonB2 protein prevents growth stimulation by catecholamines (Li et al., 2015), and in *Aeromonas hydrophila*, the increase of bacterial growth due to these molecules seems also dependent on TonB2-energy transduction system (Dong et al., 2016). Recently, Aldriwesh et al. (2019) showed that Epi and NE present in peritoneal dialysate can enhance the development of bacteria and the infection risk via transferrin iron provision. All these results showed that catecholamines can increase growth of several bacteria, and that this effect may be, in part, closely related to iron homeostasis.

**TABLE 2:** Bacterial growth in presence of catecholamines.

Gram-negative bacteria	Catecholamines	References
*Acinetobacter lwoffii, Citrobacter freundii, Enterobacter aerogenes, Enterobacter agglomerans, Enterobacter cloacae, Enterobacter sakazaki, Escherichia coli, Hafnia alvei, Klebsiella oxytoca, Klebsiella pneumoniae, Morganella morganii, Pseudomonas aeruginosa, Proteus mirabilis, Salmonella enterica sv* Enteritidis, *Serratia marcescens, Yersinia enterocolitica, Xanthomonas maltophilia*	NE	Freestone et al., 1999, 2000, 2003, Freestone, 2013
*Actinobacillus pleuropneumoniae*	Epi NE Dopamine	Li et al., 2015
*Aeromonas hydrophila*	Epi NE Dopamine	Dong et al., 2016
*Bordetella bronchiseptica*	NE	Anderson and Armstrong, 2008 Armstrong et al., 2012
*Burkholderia pseudomallei*	Epi	Intarak et al., 2014
*Campylobacter jejuni*	NE Epi NE	Cogan et al., 2007 Xu et al., 2015
*Escherichia coli*	Epi NE Dopamine	Burton et al., 2002
*Pseudomonas aeruginosa* PA14	NE NE Dopamine	Hegde et al., 2009 Freestone et al., 2012
*Pseudomonas fluorescens* MFN1032	NE	Biaggini et al., 2015
*Salmonella enterica* serovar Typhimurium	NE Dopamine	Williams et al., 2006 Dichtl et al., 2019
*Vibrio parahaemolyticus*, *Vibrio cholerae*, *Vibrio vulnificus*, Vibrio mimicus	NE	Nakano et al., 2007a
*Vibrio cholerae*	Epi NE	Halang et al., 2015
*Yersinia ruckeri*	NE Dopamine	Torabi Delshad et al., 2019

**Gram-positive bacteria**	**Catecholamines**	**References**

*Enterococcus faecalis, Enterococcus faecium, Listeria monocytogenes, Staphylococcus aureus, Staphylococcus epidermidis, Streptococcus dysgalactiae* *Listeria monocytogenes* *Listeria monocytogenes, Listeria innocua, Listeria ivanovii, Listeria seeligeri, Listeria grayi* *Staphylococcus aureus* *Staphylococcus aureus, Staphylococcus epidermidis* *Staphylococcus epidermidis* *Streptococcus mutans, Streptococcus mitis, Streptococcus gordonii, Streptococcus* *intermedius*	NE NE Epi NE Dopamine Epi NE NE NE Epi NE	Freestone et al., 1999, 2000, 2003, Freestone, 2013 Coulanges et al., 1997 Coulanges et al., 1998 Belay and Sonnenfeld, 2002 Mart’yanov et al., 2021 Lyte et al., 2003 Roberts et al., 2002

### Effect of Catecholamines on Chemotaxis and Motility

Chemotaxis is one way in which bacteria could react to the compounds present in their environment. It allows them to navigate in gradients of various chemicals in order to locate conditions that are beneficial for growth. Scarce studies are available on the effect of catecholamines on chemotaxis. Chet et al. (1973) have reported that the chemotactic response of *P. fluorescens* was significantly enhanced by Epi. Later, using an agarose plug chemotaxis assay, Bansal et al. (2007) found that both Epi and NE were chemo-attractants for *E. coli* O157:H7. More recently, Lopes and Sourjik (2018) showed that *E. coli* RP437 reacts with mixed responses for dopamine and NE using FRET (Fluorescence Resonance Energy Transfer) and microfluidics assay. Indeed, both hormones elicited biphasic results. Dopamine was sensed as a repellent at concentrations below 1 mM and as attractant at 10 mM. The response to NE had an inverse pattern. This molecule behaved as a weak attractant at low concentrations, but it produced a repellent response above 1 mM.

More data are available concerning catecholamines and motility tests (Table 3). These molecules have been found to enhance the motility of numerous bacteria, including pathogenic strains as *P. fluorescens* MFN1032 (Biaggini et al., 2015), *P. aeruginosa* PAO1/H103 (Cambronel et al., 2019), *P. aeruginosa* PA14 (Hegde et al., 2009), *S. enterica* serovar Typhimurium (Bearson and Bearson, 2008; Peterson et al., 2011), *E. coli* O157:H7 (Bansal et al., 2007), and some *Campylobacter* and *Vibrio* species (Cogan et al., 2007; Yang et al., 2021). In *V. harveyi*, the addition of the eukaryotic α-adrenergic antagonist phentolamine, or the bacterial catecholamine receptor antagonist LED209, was able to neutralize NE-induced swimming motility (Yang Q. et al., 2014). These authors found also that the dopaminergic antagonist chlorpromazine and the LED209 antagonist reduced dopamine-induced motility of this bacterium. The motility of other *Vibrio* species (*V. anguillarum*, *V. campbellii*, *V. parahaemolyticus*) can also be modulated by catecholamines (Pande et al., 2014). Chlorpromazine was able to stop the effect of dopamine in *V. anguillarum* and *V. campbellii*, and the α-adrenergic receptor antagonists phentolamine and phenoxybenzamine neutralized the effect of NE, whereas the β-adrenergic receptor antagonist propranolol had limited to no effect. This antagonist also failed to block the motility induced by NE in *Yersinia ruckeri* (Torabi Delshad et al., 2019). In *V. parahaemolyticus*, the bacterial antagonist LED209 neutralized the stimulatory effects of catecholamines on the growth and motility of the bacteria (Yang et al., 2021). In *Burkholderia pseudomallei*, phentolamine was found to reverse only partially the effect of Epi on motility (Intarak et al., 2014).

**TABLE 3:** Bacterial motility and chemotaxis in presence of catecholamines.

Bacterial species	Catecholamines (dose)	Antagonists	References
*Burkholderia pseudomallei* *Campylobacter jejuni NCTC11168* *Escherichia coli* O157:H7 *Escherichia coli* K12-MC1000 *Pseudomonas aeruginosa* PAO1 (H103) *Pseudomonas aeruginosa* PA14 *Pseudomonas fluorescens MFN1032*	Epi (50 μM) NE (100 μM) Epi, NE (100 μM) Epi, NE (50 μM) Epi, NE (50 μM) Epi (1–10 μM) NE (50–500 μM) Epi, NE, Dopamine Epi (10 μM)	Phentolamine n. n. n. n. n.	Intarak et al., 2014 Cogan et al., 2007 Xu et al., 2015 Bansal et al., 2007 Yang K. et al., 2014 Cambronel et al., 2019 Hegde et al., 2009 Freestone et al., 2012 Biaggini et al., 2015
*Salmonella enterica* serovar Typhimurium *Vibrio anguillarum* *Vibrio campbellii*	NE (50 μM) NE (100 μM) Dopamine (100 μM)	Phentolamine Phentolamine Phenoxybenzamine Propanolol Chlorpromazine	Bearson and Bearson, 2008 Peterson et al., 2011 Pande et al., 2014
*Vibrio harveyi (campbellii)*	NE (50 μM)	Phentolamine Phenoxybenzamine Labetalol Propanolol LED209	Yang Q. et al., 2014
	Dopamine (50 μM)	Chlorpromazine LED209	
*Vibrio parahaemolyticus*	Epi (100 μM NE (50 μM) Dopamine (50 μM)	LED209	Yang et al., 2021
*Yersinia ruckeri*	NE (100 μM) Dopamine (100 μM)	Propanolol Labetalol Phenoxybenzamine Chlorpromazine	Torabi Delshad et al., 2019

*n, not used in the study.*

The expression of genes involved in flagellum formation and motility has been quantified in some bacteria exposed to catecholamines, and thus found to be modulated. Indeed, Yang Q. et al. (2014) showed that NE and Dopa significantly up-regulate the expression of ten genes involved in the flagella synthesis and chemotaxis of *V. harveyi*. The same result was observed for *C. jejuni* exposed to Epi and NE, in which many of the up-regulated genes were involved in flagellar assembly pathway (Xu et al., 2015). Surprisingly, in *P. aeruginosa* PA14, a treatment with 50 μM NE was found to decrease the expression of motility genes, while at 500 μM, they were up-regulated (Hegde et al., 2009), showing the influence of the concentration of stress hormones on their effects.

Thus, the presence of Epi or NE has been shown in multiple investigations to significantly enhance the motility of bacteria. However, an impact on the transcription of genes of the flagellar or chemotaxis operons is not always apparent, probably due to the experimental conditions. In fact, gene expression assays are usually quantified using broth cultures whereas motility assays are typically performed using semi-solid agar medium. The differences in incubation conditions for growth rate and growth phase in broth and motility assays may account for a lack of congruence between transcriptional analysis and motility phenotype.

### Effect on Biofilm

Biofilms are composed of cells bound to a surface and to each other and embedded within a matrix of extracellular polymeric substances they have produced (Donlan, 2002). For most of the microorganisms, the ability to form biofilms is a key factor to colonize and survive in the host environment (Costerton et al., 1999; Donlan and Costerton, 2002), promoting bacterial growth and pathogenicity (Parsek and Singh, 2003). Catecholamines were found to stimulate the biofilm in several bacteria (Table 4). First evidences of positive effect were obtained with utilization of the catecholamines inotropes, NE, and dobutamine, on *S. epidermidis* (Lyte et al., 2003). These authors found that incubation of this bacterium with catecholamines in the presence of human plasma resulted in a significant increase of growth and biofilm formation on both polystyrene and silicone surfaces, which was associated with extensive exopolysaccharide production. This suggested that the stimulation of bacterial proliferation and biofilm formation by these drugs may be an etiological factor in the development of intravascular catheter colonization and catheter-related infection. Since then, several other studies have shown an impact of catecholamines on adhesion and biofilm formation for various bacteria. Most of these experiments have also been conducted on abiotic surfaces. For example, Yang Q. et al. (2014) demonstrated that both NE and dopamine could increase the biofilm formation and exopolysaccharides production in *V. harveyi*, and this was blocked by the antagonists phentolamine, phenoxybenzamine, labetalol and LED209 for NE, chlorpromazine and LED209 for dopamine. Similarly, the biofilm formation of *Y. ruckeri* was found to be enhanced by NE and dopamine and antagonists were used to inhibit these effects (Torabi Delshad et al., 2019). In *E. coli* K-12 MC1000, addition of Epi/NE increased biofilm’s thickness on polyvinyl chloride surface through the QseC sensor (Yang K. et al., 2014). Siderophores and ferric iron transport system also appear to play a vital role in the mechanism by which catecholamines influence biofilms formation (Feraco et al., 2016). In *P. aeruginosa* H103, a flow-cell device with glass surface was used to investigate the biofilm formation in dynamic conditions in the presence of Epi (Cambronel et al., 2019). The authors found no modification of the biofilm architecture with this catecholamine, but thicknesses and biovolume were significantly increased with 10 μM Epi compared to the untreated biofilm. Interestingly, it has been demonstrated that catecholamines had no impact on the growth of the Gram-positive *Cutibacterium acnes*, but were able to promote its biofilm formation (Borrel et al., 2019). Recently, the effect of catecholamines were also investigated in *E. faecalis* and the authors showed that Epi and NE can stimulate biofilm formation and adhesion of pathogenic but also probiotic strains of this species (Cambronel et al., 2020). On the contrary, the responses of *S. lugdunensis*, *S. epidermidis*, and *S. aureus* to Epi and dopamine were more variable, with increase or decrease of biofilm formation depending on the tested strains (Frank and Patel, 2008). Moreover, Epi and NE were found to promote the dispersion of biofilm in *Mannheimia haemolytica* (Pillai et al., 2018). Similarly, Lanter and Davies (2015) demonstrated that *C. acnes* biofilms were dispersed when challenged with NE in the presence of iron-bound transferrin or with free iron. Another study by Lanter et al. (2014) also showed that addition of NE (at 400 μM) induced dispersion of *P. aeruginosa* biofilms when grown under low iron conditions in the presence of transferrin. This dispersion is related to the release of degradation enzymes that can be harmful to the host tissue (Lanter et al., 2014).

**TABLE 4:** Bacterial biofilm in presence of catecholamines.

Bacterial species	Catecholamines (dose)	Effects on biofilms	Antagonists	References
*Aeromonas hydrophila*	Epi (100 μM) NE (100 μM) Dopamine (100 μM)	Increase (Crystal violet staining)	n.	Dong et al., 2016
*Cutibacterium acnes*	Epi (1 μM) NE (1 μM)	Increase (Crystal violet staining and CLSM analyses)	n.	Borrel et al., 2019
*Cutibacterium acnes* VP1	NE (400 μM)	Dispersion (Quantification of bacteria released from biofilm)	n.	Lanter and Davies, 2015
*Enterococcus faecalis*	Epi (1–100 μM) NE (1–100 μM)	Increase (Crystal violet staining and CLSM analyses)	n.	Cambronel et al., 2020
*Escherichia coli* K12-MC1000	Epi (50 μM) NE (50 μM)	Biofilm increase (Crystal violet staining, CLSM and SEM analyses)	n.	Yang K. et al., 2014
*Escherichia coli* O157:H7	Epi (50 μM) NE (50 μM)	Biofilm increase (Crystal violet staining, microarray)	n.	Bansal et al., 2007
*Mannheimia haemolytica*	Epi (50 μM) NE (50 μM)	Dispersion (Crystal violet staining, CLSM and SEM analyses)	n.	Pillai et al., 2018
*Pseudomonas aeruginosa* H103	Epi (1–10 μM)	Increase (CLSM analyses)	n.	Cambronel et al., 2019
*Pseudomonas aeruginosa* PAO1	NE (400 μM)	Dispersion (Quantification of bacteria released from biofilm)	n.	Lanter et al., 2014
*Pseudomonas aeruginosa* PA14	Epi (5 μM) NE (5 μM) Dopamine (5 μM)	Increase (Crystal violet staining, SEM analyses)	n.	Freestone et al., 2012
*Staphylococcus epidermidis*	NE (100 μM)	Increase (SEM analyses)	n.	Lyte et al., 2003
*Staphylococcus lugdunensis Staphylococcus epidermidis Staphylococcus aureus*	Epi (0–50 μg/mL) Dopamine (0–32 μg/mL)	Increase or decrease (Safranin staining and quantification)	n.	Frank and Patel, 2008
*Staphylococcus aureus Staphylococcus epidermidis* (dual species biofilm)	NE (10^–7^M)	Enumeration on Luria-Bertani agar plates	n.	Mart’yanov et al., 2021
*Vibrio harveyi (Campbellii)*	NE (50 μM) Dopamine (50 μM)	Increase (Crystal violet staining)	Phentolamine LED209 Phenoxybenzamine Labetalol Propranolol	Yang Q. et al., 2014
*Yersinia ruckeri*	NE (100 μM) Dopamine (100 μM)	Increase (Crystal violet staining)	Chlorpromazine Phenoxybenzamine Labetalol Propranolol Chlorpromazine	Torabi Delshad et al., 2019

*n, not used in the study; CLSM, confocal laser scanning microscope; SEM, scanning electron microscopy.*

The impact of catecholamines on mixed biofilms remains to be investigated in the future, to better understand the competition between bacteria in ecological niches. A recent publication explored for the first time the impact of NE on monospecies and dual-species biofilms of *S. epidermidis* and *S. aureus* (Mart’yanov et al., 2021). These authors showed that NE can affect the biofilm formation of both species with a strong dependence on aerobic or anaerobic culture conditions. They found that *S. epidermidis* suppresses *S. aureus* growth in dual-species biofilms and that NE can accelerate this process, contributing to the competitive behavior of staphylococci.

### Effect on Adhesion/Invasion, Cytotoxicity and Virulence

Several studies have investigated the impact of catecholamines on the interaction between bacteria and eukaryotic cells (Table 5). NE or Epi were found to be able to stimulate the adhesion potential of bacteria (e.g., *A. hydrophyla, Act. pleuropneumoniae, C. jejuni, E. faecalis, P. aeruginosa*) on lung and intestinal cells. Conversely, a decrease of adhesion has been observed for *Streptococcus pneumoniae* on A549 lung cells when the bacteria were pre-treated with NE or incubated with cells in presence of NE (Gonzales et al., 2013). The cytotoxic activity of catecholamines have been studied in a porcine lung epithelial cell line (SJPL), infected by *Act. pleuropneumoniae* (Li et al., 2012), using a CytoTox 96 LDH kit, and staining with crystal violet. Both methods showed that cytolytic activity was significantly increased by Epi but repressed by NE. The adrenergic receptor antagonists phentolamine and propanolol were found to be able to reverse significantly the effects of Epi and NE. In a study conducted by Biaggini et al. (2015), *P. fluorescens* treated with Epi led to 75% mortality of the undifferentiated Caco-2/TC7 cells, but the cytotoxicity of the untreated bacteria was almost the same with about 70% cell mortality. Nakano et al. (2007b) have shown that NE changed expression of TTSS1-related genes of *V. parahaemolyticus* and induced cytotoxic activity.

**TABLE 5:** *In vitro* bacterial effects in presence of catecholamines (adhesion/invasion, cytotoxicity).

Bacterial species	Catecholamines (dose)	Cell lines	*In vitro* effects	Antagonists	References
*Aeromonas hydrophyla*	Epi (100 μM) NE (100 μM) Dopamine (100 μM)	HEp-2 epithelial cells	Increase of adhesion	n.	Dong et al., 2016
*Actinobacillus pleuropneumoniae*	Epi (50 μM) NE (50 μM)	SJPL lung cells	Adhesion induced by NE but not by Epi Cytotoxicity enhanced by Epi but repressed by NE	Phentolamine Propanolol	Li et al., 2012
*Campylobacter jejuni* NCTC11168	NE (100 μM)	Caco-2 intestinal cells	Increase of invasion Decrease of TEER Breakdown of tight junction (occludin) observed by CLSM	n.	Cogan et al., 2007
	Epi (100 μM) NE (100 μM)	Caco-2 intestinal cells	Increase of adhesion/invasion	n.	Xu et al., 2015
*Campylobacter* species	NE (100 μM)	T84 epithelial cells	Increase of invasion for *C. jejuni* M1 and *C. fetus fetus*, not for *C. jejuni* 81116 and *C. coli* 1669 Decrease of TEER breakdown of tight junction (occludin) observed by CLSM	n.	Aroori et al., 2014
*Enterococcus faecalis*	Epi (1 μM) NE (1 μM)	Caco-2/TC7 intestinal cells HaCaT keratinocyte cells	Increase of adhesion	n.	Cambronel et al., 2020
*Salmonella enterica* serovar Typhimurium	NE (50 μM)	HeLa epithelial cells	Increase of invasion	n.	Moreira and Sperandio, 2012
*Streptococcus pneumoniae*	NE (50 μM)	A549 lung cells	Decrease of adhesion	n.	Gonzales et al., 2013
*Pseudomonas aeruginosa* PAO1	Epi (1 μM)	Caco-2/TC7 intestinal cells	Increase of adhesion/invasion and translocation Decrease in TEER	n.	Biaggini, 2015
*Pseudomonas aeruginosa* PA14	NE (50 and 500 μM)	HCT-8 intestinal cells	Increase of adhesion/invasion	n.	Hegde et al., 2009
*Pseudomonas fluorescens* MFN1032	Epi (1 μM)	Caco-2/TC7 intestinal cells	No effect on cytotoxicity Decrease of TEER F-actin cytoskeleton disorganization (CLSM observation)	n.	Biaggini et al., 2015
*Vibrio parahaemolyticus*	NE (50 μM)	Caco-2 intestinal cells	Increase of cytotoxicity	Phentolamine Propanonol	Nakano et al., 2007b

*n, not used in the study; TEER, transepithelial electrical resistance; CLSM, confocal laser scanning microscope.*

In *Campylobacter*, Cogan et al. (2007) examined invasion of Caco-2 cells with *C. jejuni* NCTC11168, and noticed that at least 10 times more bacteria were recovered within epithelial cells after 2 h, when they were pretreated with NE. Later, in 2014, the same research group investigated the invasion of other *Campylobacter* strains in T84 epithelial cells, and they found that *C. jejuni* M1 and *C. fetus* subsp. *fetus* 10842 were also more invasive after 48 h pretreatment with NE, contrary to the invasion potential of *C. jejuni* subsp. *jejuni* 81116 and *C. coli* 1669 that were not modified (Aroori et al., 2014). In this study, the authors also observed that the presence of NE enhanced the reduction of transepithelial resistance (TER) of T84 cells infected by *C. jejuni* M1, *C. coli* and *C. fetus subsp. fetus* 10842; and increased breakdown of tight junctions. Similarly, experiments performed on differentiated Caco-2/TC7 cells with *P. aeruginosa* PAO1 showed that a pre-treatment of this bacterium with Epi increased its invasive and translocation potential, and the reduction of TER (Biaggini, 2015). In *Salmonella*, the presence of 50 μM Epi in a late log phase LB-culture enhanced by 1.5-fold its invasion of HeLa cells, with an evident impact of QseC and QseE on the pathogenicity island 1 (SPI-1) upregulation (increase of *sopB* and *sipA* transcription by two-fold) that is involved in the invasion phenotype (Moreira and Sperandio, 2012). Similar effect was observed with NE in the same growth conditions, where *invF* and *sopB* encoded in SPI-1 were increased by >15-fold. In contrast, Karavolos et al. (2008) indicated a downregulation of *invF* following 30 min of exposure to 50 μM Epi in similar growth conditions.

Few *in vivo* experiments were also conducted by some researchers (Table 6). Pande et al. (2014) showed that 100 μM dopamine and NE significantly increased the virulence of *V. campbellii* BB120 in a model of giant shrimp larvae. The survival of the larvae was found to be reduced by 15%, after 6–8 days of infection with dopamine or NE-pretreated *V. campbellii* BB120, compared to infection with untreated bacteria. In this work, a dopaminergic receptor antagonist for dopamine (chlorpromazine) and an adrenergic receptor antagonist (phentolamine), were also tested. The authors found that chlorpromazine could not neutralize the effect of dopamine, whereas the adrenergic receptor antagonist phentolamine only neutralized the effect of NE at a relatively high concentration (500 μM) and in only 2 of the 3 trials. Similarly, in *Y. ruckeri*, these catecholamines (dopamine and NE) significantly enhanced the virulence towards rainbow trout, and some antagonists were able to neutralize the effect of the stress hormones (Torabi Delshad et al., 2019). Indeed, phenoxybenzamine (the α-adrenergic antagonist) and labetolol (the α- and β-adrenergic antagonist) were able to block the increased virulence induced by NE, and chlorpromazine (the dopaminergic antagonist) could inhibit the effect of dopamine. On the contrary, propranolol did not show any antagonist ability to neutralize the effect of NE. In a mouse model, Dong et al. (2016) found that NE increased the proliferation capacity of *A. hydrophila* in the lungs. Initially administered in the intestine, the bacteria translocated through the gut and were able to disseminate into the lungs of mice. Another study conducted by Pullinger et al. (2010) found that NE augments *Salmonella enterica*-induced enteritis in pigs, in association with increased net replication but independent of the putative adrenergic sensor kinases QseC and QseE. Gao et al. (2019) found that the mortality of crucian carp challenged with *A. hydrophila* AH196 was significantly higher in the group treated with NE. In the same study, real-time PCR analyses revealed that NE notably up-regulated 13 out of 26 virulence-associated genes expression. Dowd (2007) reported that NE increased the expression levels of the shiga-toxins coding-genes *stx1* and *stx2* of *E. coli* O157:H7 during the 5 h incubation.

**TABLE 6:** *In vivo* bacterial effects in presence of catecholamines.

Bacterial species	Catecholamines (dose)	*In vivo* effects	Antagonists	References
*Aeromonas hydrophyla*	NE (1 mg/400 μL)	NE enhances the systemic spread of the bacteria during infection of mice	n.	Dong et al., 2016
	NE (100 μM)	Increased mortality of crucian carp	n.	Gao et al., 2019
*Pseudomonas aeruginosa* H103	Epi (1–10 μM)	Increased mortality of *Galleria mellonella* larvae (with 10 μM Epi)	n.	Cambronel et al., 2019
*Salmonella enterica* serovar Typhimurium	NE (200 mg/kg)	Oral administration of NE, but not preculture with NE alters the course of infection in pigs	n.	Pullinger et al., 2010
*Vibrio campbelli* BB120	NE/Dopamine (100 μM)	Increased virulence toward giant freshwater prawn larvae (*Macrobrachium rosenbergii*)	Phentolamine Chlorpromazine	Pande et al., 2014
*Vibrio harveyi*	NE/Dopamine (50 μM)	Increased virulence toward gnotobiotic brine shrimp larvae	Phentolamine Phenoxybenzamine Labetalol LED209 Chlorpromazine	Yang Q. et al., 2014
*Yersinia ruckeri*	NE/Dopamine (100 μM)	Increased mortality of rainbow trout	Phenoxybenzamine Labetalol Propanolol Chlorpromazine	Torabi Delshad et al., 2019

*n, not used in the study.*

### Other Effects

Catecholamines have been found to be able to modulate the sensitivity/resistance to antibiotics. For example, it has been shown that catecholamines can help bacteria for better growth recovery following antibiotic treatment (Freestone et al., 2012). Indeed, when *P. aeruginosa* PA14 was exposed to sub-inhibitory concentrations of tobramycin in serum-SAPI medium, the authors showed that adding NE allowed the bacteria to have a better growth (10^3^ CFU/mL at 24 h versus 5.10^5^ CFU/mL with NE). This effect has also been observed in *S. epidermidis* (Freestone et al., 2016) and *A. baumannii* (Inaba et al., 2016). In *C. jejuni*, transcriptomic analysis showed that two genes involved in antimicrobial peptide resistance (Cj1583c and Cj0193c) were overexpressed in the presence of 100 μM Epi or NE (Xu et al., 2015). On the contrary, in *S. enterica* serovar Typhimurium, Epi reduced the ability of the bacteria to survive polymyxin B treatment (Karavolos et al., 2008). Using microarray analysis, the authors observed a decrease in the expression of the *pmr* regulon (*pmrHFIJKLM*, responsible for polymyxin resistance), which was mediated by the BasSR two component signal transduction system. This phenotype was reversed by the addition of the β-adrenergic blocker propranolol. Given that PmrAB activation was observed in the presence of extracellular iron for prevention of iron toxicity (Wösten et al., 2000), the binding of iron by Epi may partly explain the decreased expression of the PmrAB regulon. Karavolos et al. (2008) also showed that Epi increased oxidative stress resistance in *S. enterica* serovar Typhimurium when the bacterium was exposed to the molecule. Indeed, using transcriptomic assay and a luminescent transcriptional reporter, the authors found that the superoxide dismutase *sodA* gene was significantly upregulated by Epi. Similarly, Intarak et al. (2014) found that Epi-treated *Burkholderia pseudomallei* exhibited increased resistance to superoxide, consistent with induction of *sod*B expression.

Besides those effects, catecholamines may also regulate the production of some metabolites. At the human oral microbiome, production of volatile sulfur compounds, the major gasses responsible for bad breath (mainly hydrogen sulfide H_2_S) was observed in periodontal pathogenic bacteria (i.e., *Fusobacterium nucleatum*, *Porphyromonas endodontalis*, *Prevotella intermedia*, and *Porphyromonas*) exposed to catecholamines (Roberts et al., 2002; Calil et al., 2014; de Lima et al., 2018).

Horizontal gene transfer (HGT) in bacteria is another mechanism impacted by host signals with an enhanced conjugative gene transfer observed between enteric bacteria (Peterson et al., 2011). Exposure to 5 μM NE in LB medium stimulated the transfer of a conjugative plasmid encoding multidrug resistance from a clinical *S.* Typhimurium strain to a recipient *E. coli* strain. Interestingly, a significant up-regulation of *tra* gene expression involved with plasmid transfer was observed. Treatment with the adrenergic receptor antagonists (phentolamine at 500 μM) negated the NE-enhanced conjugation frequency to baseline levels. Thus, these mediators of host stress may influence the evolution and adaptation of pathogens in the environment due to the transfer of genes that encode resistance to antibiotics and virulence factors.

### Meta-Effects Studied by Transcriptomic/Proteomic Analyses

The global effects of catecholamines on bacteria have been first studied on a *luxS* enterohemorrhagic *E. coli* (EHEC) mutant by Kendall et al. (2007) using 50 μM of Epi and GeneChip microarrays (Affymetric system) for transcriptome analysis (Table 7). The authors observed a differential expression for 5,204 genes. In fact, Epi mainly increased the expression of the LEE regulon, which is known to play a pivotal role in EHEC virulence. The activated genes included the LEE genes, the flagellar regulon genes, the genes encoding iron uptake systems, the gene encoding the Hfq protein (a chaperone involved in small regulatory RNA post-transcriptional regulation), and genes encoding several nucleoid proteins, all reported to be involved in LEE regulation.

**TABLE 7:** Meta-effects of catecholamines on bacteria studied by transcriptomic analysis.

Bacterial species	Catecholamines (dose)	Meta-effects	References
*Actinobacillus pleuropneumoniae luxS Escherichia coli* EHEC mutant	Epi (50 μM) NE (100 μM) Epi (50 μM)	Differential expression of 158 and 105 genes, for Epi and NE, respectively. Many virulence factors. Only 18 genes regulated by both hormones Differential expression of 5,204 genes. Increased expression of the locus of enterocyte effacement (LEE), flagellar regulon genes, iron uptake systems	Li et al., 2012 Kendall et al., 2007
*Salmonella enterica* serovar Typhimurium	Epi (50 μM)	Modulation of 0.6% of the transcriptome. Upregulation of genes involved in metal homeostasis and oxidative stress	Karavolos et al., 2008
*Pseudomonas aeruginosa* PA14	NE (50–500 μM)	Exposure to 50 μM NE altered the expression of 184 genes (128 induced, 56 repressed) Exposure to 500 μM NE induced 287 genes and repressed 50 genes. Up-regulation of virulence with 500 μM NE but not 50	Hegde et al., 2009
*Campylobacter jejuni* NCTC11168	Epi (100 μM) NE (100 μM)	Differential expression of 183 and 156 genes, for Epi and NE, respectively. 102 of these modulated genes were common for the two hormones (iron uptake, motility, virulence, oxidative stress response, nitrosative stress tolerance, enzyme metabolism, DNA repair and metabolism, ribosomal protein biosynthesis)	Xu et al., 2015

Another publication reported the global effects of Epi in *S. enterica* serovar Typhimurium (Karavolos et al., 2008). In this work, the transcriptomic analyses showed that approximately 0.6% of the transcriptome of the pathogen was significantly regulated by 50 μM Epi. The major feature of this bacterial adrenaline response was the upregulation of genes involved in metal homeostasis and oxidative stress. The key metal transport systems were induced within 30 min of treatment. Alterations in genes encoding proteins of unknown functions and changes in levels of regulators and signal transduction genes were also noticed.

Hegde et al. (2009) analyzed the transcriptome of *P. aeruginosa* PA14 exposed to 50 and 500 μM NE for 7 h. They found that 500 μM but not 50 μM of this molecule upregulated the genes involved in the regulation of the virulence determinants pyocyanin, elastase, and the *Pseudomonas* quinolone signal (PQS, 2-heptyl-3-hydroxy-4-quinolone).

Genome-wide Mu*d*J transposon mutagenesis was used to study Epi- and NE-regulated genes in *S. Typhimurium* (Spencer et al., 2010). A transposon library of 10,000 *S.* Typhimurium mutants was screened and led to the identification of seven down-regulated and one up-regulated fusions in the presence of 250 μM Epi. The down-regulated genes included two virulence-related genes *virK* and *mig14* (involved in bacterial resistance to antimicrobial peptides), in addition to *iroC* (ABC transporter), *accC* (Acetyl-CoA carboxylase subunit), *nrdF* (Ribonucleotide diphosphate reductase subunit), *yedP* (Putative mannosyl-3-phosphoglycerate phosphatase), and STM3081 (Putative l-lactate/malate dehydrogenase), while the *yhaK* gene was up-regulated whose product is a putative cytoplasmic protein of unknown function. The regulation of *virK*, *mig14* and *yhaK* by 500 μM Epi and NE could be reversed in a promoter-luciferase fusion assay by addition of an α-adrenergic antagonist (phentolamine at 500 μM). In addition, exposure to 500 μM Epi or NE significantly increased sensitivity of *S.* Typhimurium to the antimicrobial peptide cathelicidin LL-37. Interestingly, a significant increase in sensitivity to LL-37 was demonstrated for the *virK* mutant in the absence of catecholamines.

Later, Li et al. (2012) investigated the global effects of catecholamines on *Act. pleuropneumoniae*, an important porcine respiratory pathogen causing significant economic loss in the global pig industry. Gene expression profiles after Epi and NE treatment were compared with untreated bacteria. The microarray results showed that 158 and 105 genes were differentially expressed in the presence of Epi and NE, respectively. These genes were assigned to various functional categories including many virulence factors, whereas only 18 genes were regulated by both catecholamines. Thus, differential regulation of gene expression suggests that this pathogen may have multiple responsive systems for the two hormones.

Transcriptomic analyses using Agilent microarrays were then performed in *C. jejuni* NCTC 11168, cultivated in iron-restricted medium, and supplemented with Epi and NE (Xu et al., 2015). The authors found that Epi and NE respectively modified the expression of 183 and 156 genes, compared to the expression in absence of these hormones, and 102 of these modulated genes were common for Epi and NE treatments. Various cellular functions were found to be modified, including iron uptake, motility, virulence, oxidative stress response, nitrosative stress tolerance, enzyme metabolism, DNA repair and metabolism, and ribosomal protein biosynthesis.

At the time of writing this review, only two proteomic analyses on the effect of catecholamines on bacteria were retrieved. The first study was done by Toulouse et al. (2019). The authors investigated the impact of Epi and adrenochrome on *V. cholerae*. They observed a proteome change in proteins involved in iron homeostasis, metabolism, signaling or translational and transcriptional control. The second study was conducted by Scardaci et al. (2021) using the probiotic strain *Enterococcus faecium* NCIMB10415. Combining a gel-free/label-free proteomic analysis, these authors evaluated the global changes induced by NE treatment in the bacterium. They found that exposure of *E. faecium* NCIMB10415 to this bioactive molecule enhanced the abundance of proteins related to stress response and to host-microbe interaction, such as moonlight proteins involved in adhesion and immune stimulation.

All these meta-effects showed that catecholamines can modulate several functions in bacteria, but it is important to notice that the results obtained may depend on the hormone (Epi or NE), their concentrations, and the medium used for the bacterial growth.

## Conclusion and Future Prospect

Thirty years of studies in the field of microbial endocrinology have shown that there are multiple bi-directional interactions between bacteria and their host. In such interactions, the main actors are neurotransmitters whose mission is the transmission of information within and between living cells, giving the inter-kingdom signaling. As for the genetic code shared by all living organisms, a common class of mediator-type molecules indicates that the mechanism of communication within neurotransmitters appears to be widely recognized. The role of regulatory proteins in catecholamines sensing is an area of growing research. Though evidence exists that catecholamines exert effects on bacteria within *in vitro* culture conditions (i.e., growth, motility, adhesion, virulence). However, these effects are mainly dependent on media composition and inoculum density (growth phase), being most prominent in a minimal salt’s medium (i.e., serum-SAPI) miming nutrient poor and iron-limited conditions encountered *in vivo*. The variability of results obtained in the literature concerning catecholamines can also be due to a poor stability of the molecules in solution as they need to be freshly prepared before the experiments.

Within the human microbiome, the ability of microorganisms to respond to the panoply of neuroendocrine hormones is becoming increasingly recognized as playing a pivotal role in disease pathogenesis and maintenance of homeostasis (Lyte, 2013). In the near future, new methodologies as organ-on-chip will be a promising sophisticated tool to investigate the interactions between bacteria and the host in the presence of several metabolites and molecules including hormones. This state of art technology will ensure a stable and/or variable dynamic flow of catecholamines, on bacteria and eukaryotic cells, and thus simulates more accurately what can happen *in vivo*, especially in case of a stress peak.

During their evolution, pathogenic bacteria can adapt and develop sophisticated systems allowing them to sense eukaryotic signals and to use them to their advantage to stimulate their colonization and virulence. While several data indicate that Gram-negative bacterial pathogens possess elements that specifically interact with catecholamines allowing them to sense the host environment, scarce data is available on such elements found in Gram-positive bacteria and especially in probiotic bacteria. A better knowledge of these systems will allow to decipher how stress hormones could be involved in the colonization by microbiota and in the eubiosis/dysbiosis of the gut or other organs. This may help to develop new treatments with medical and economic interest.

## Author Contributions

NC supervised the project of the review. All authors participated in the redaction of the manuscript and accepted the final version.

## Conflict of Interest

The authors declare that the research was conducted in the absence of any commercial or financial relationships that could be construed as a potential conflict of interest.

## Publisher’s Note

All claims expressed in this article are solely those of the authors and do not necessarily represent those of their affiliated organizations, or those of the publisher, the editors and the reviewers. Any product that may be evaluated in this article, or claim that may be made by its manufacturer, is not guaranteed or endorsed by the publisher.
